# The 2007 Los Angeles Mommy and Baby Study: A Multilevel, Population-Based Study of Maternal and Infant Health in Los Angeles County

**DOI:** 10.1155/2014/293648

**Published:** 2014-12-11

**Authors:** Shin M. Chao, Fathima Wakeel, Dena Herman, Chandra Higgins, Lu Shi, Jessica Chow, Stacy Sun, Michael C. Lu

**Affiliations:** ^1^Research, Evaluation and Planning Division, Los Angeles County Department of Maternal, Child and Adolescent Health Programs, 600 Commonwealth Avenue, 8th Floor, Los Angeles, CA 90095, USA; ^2^Ferris State University College of Health Professions, 200 Ferris Drive, VFS 428, Big Rapids, MI 49307, USA; ^3^Department of Family and Consumer Sciences, California State University, Northridge, 18111 Nordhoff Street, Northridge, CA 91330, USA; ^4^Clemson University Department of Public Health Sciences, 505 Edwards Hall, Clemson, SC 29634, USA; ^5^University of California, Berkeley-San Francisco Joint Medical Program, 50 University Hall, No. 7360, San Francisco, CA 94720, USA; ^6^Department of Gynecology and Obstetrics, Johns Hopkins University School of Medicine, 600 North Wolfe Street, Phipps 279, Baltimore, MD 21287, USA; ^7^University of California, Los Angeles (UCLA) School of Public Health, 650 Charles E. Young Dr. South, Los Angeles, CA 90024, USA

## Abstract

*Objectives*. In order to comprehensively examine the risks and resources associated with racial-ethnic disparities in adverse obstetric outcomes, the Los Angeles County Department of Public Health and the University of California, Los Angeles, joined efforts to design and implement the 2007 Los Angeles Mommy and Baby (LAMB) study. This paper aims to present the conceptual frameworks underlying the study's development, highlight the successful collaboration between a research institution and local health department, describe the distinguishing characteristics of its methodology, and discuss the study's implications for research, programs, and policies. *Methods*. The LAMB study utilized a multilevel, multistage cluster design with a mixed-mode methodology for data collection. Two samples were ultimately produced: the multilevel sample (*n* = 4,518) and the augmented final sample (*n* = 6,264). *Results*. The LAMB study allowed us to collect multilevel data on the risks and resources associated with racial-ethnic disparities in adverse obstetric outcomes. Both samples were more likely to be Hispanic, aged 20–34 years, completed at least 12 years of schooling, and spoke English. *Conclusions*. The LAMB study represents the successful collaboration between an academic institution and local health department and is a theoretically based research database and surveillance system that informs effective programmatic and policy interventions to improve outcomes among LAC's varied demographic groups.

## 1. Introduction 

In Los Angeles County (LAC), one of the most populous and diverse counties in the country [1–3], there are significant geographic and racial-ethnic disparities in the prevalence of infant mortality and adverse obstetric outcomes, specifically low birth weight (LBW) and preterm birth (PTB) [3]. Historically, the LAC Department of Public Health (LACDPH) Maternal, Child and Adolescent Health (MCAH) Programs lacked surveillance data on LAC mothers. MCAH used vital records data to monitor infant health, implement programs, and plan services. The data, however, did not identify the multifaceted reasons for differences across groups or county regions. The statewide Maternal and Infant Health Assessment (MIHA) [4], California's version of the Pregnancy Risk Assessment Monitoring Study (PRAMS) [5], also focused primarily on individual prenatal risk behaviors and did not lend itself methodologically to multilevel analyses. In addition, greater flexibility was needed to assess the complex causes of LBW and PTB in LAC.

Therefore, in 2006, a collaborative effort was formed between LACDPH and the University of California, Los Angeles (UCLA) to design and implement the 2007 Los Angeles Mommy and Baby (LAMB) study, a multilevel, cross-sectional, population-based study of mothers who had a live birth in LAC in 2007. This study was developed as an important first step to expand the public health core function of assessment to include not only vital statistics, but multilevel, life-course determinants of LBW and PTB as well. The distinguishing feature of the LAMB study was that, unlike previous studies, it allowed for routine surveillance and multilevel analyses of the contextual determinants of LBW and PTB, which have been recognized in the literature as the underlying causes of these outcomes. The aims of the 2007 LAMB study included (1) an expanded assessment of racial-ethnic disparities in LBW and PTB; (2) innovative sampling methods, survey questions, and analytic techniques that allow for multilevel analyses; and (3) an academic-public health partnership that built on previously identified study questions and hypotheses driven by our collaborative work in community-based participatory research.

LBW and PTB are the leading causes of racial-ethnic disparities in perinatal mortality and morbidity in the United States and in LAC. Causes for the persisting disparities in LBW and PTB remain largely unexplained. Most extant studies, such as PRAMS, focus on individual biomedical and behavioral risk factors [6–9], which do not adequately account for racial-ethnic gaps in adverse obstetric outcomes [9–11]. In recent years, a small but growing number of studies have begun to examine obstetric outcomes and other maternal and child health (MCH) outcomes in terms of social determinants of health, including family support and violence [12–14], neighborhood characteristics such as poverty [15–17], housing [15], unemployment [17], safety [18], social capital [19], institutional environments such as working conditions [20], healthcare practices [21], cultural norms and acculturation [22–24], and racism manifested as interpersonal discrimination [25–28] or residential segregation [29, 30]. These social factors may contribute to racial-ethnic disparities in obstetric outcomes that are above and beyond individual differences. Thus, researchers have been calling for more contextual and longitudinal integration in perinatal health research and interventions [31, 32]. Therefore, this paper presents the integrated conceptual framework based on the life-course and ecological theories underlying the 2007 LAMB study, highlights the unique characteristics of its sample and methodology, and discusses the implications of this study for research, programs, and policies.

In order to comprehensively examine the determinants of racial and ethnic disparities in LBW and PTB, the development of the 2007 LAMB study was guided by two emerging conceptual frameworks, namely, the ecological model [33, 34] and the life-course perspective [35–37]. The ecological model posits that racial-ethnic disparities in adverse obstetric outcomes arise from not one single risk factor but rather from differential exposures to risks and resources from multiple sources (e.g., individual, interpersonal, neighborhood and community, institutional, and policy sources) that pattern women's biological, psychological, and behavioral responses during pregnancy [31]. In order to empirically apply the ecological theory and allow for multilevel analyses, the LAMB study constructed a multilevel sample of births in LAC, derived from sampling within census tracts and then within neighborhoods. The life-course perspective views obstetric outcomes as the product of not only the nine months of pregnancy, but the entire life-course of the mother (and father) as well. Disparities in obstetric outcomes, therefore, are the consequences of not only differential prenatal exposures, but also differential experiences before, during, and between pregnancies and across the life span [31].

## 2. Methods

### 2.1. Sample

LAC residents who delivered a live birth in 2007 were eligible to participate and were recruited 4–7 months after a live birth. For twins or triplets, one baby was randomly selected. For public health planning purposes, LAC is divided into eight geographically distinct Service Planning Areas (SPAs).

The 2007 LAMB study was designed as a multilevel survey as well as a surveillance tool. The sampling process involved three stages: two stages to identify the sample for the multilevel study and the third stage to supplement cases for county surveillance. First, we sampled neighborhoods based on census tracts and then sampled births within these neighborhoods [52]. Specifically, census tracts in LAC were divided into two strata (i.e., high-risk and low-risk), based on six perinatal indicators (i.e., number and proportion of women of reproductive age living with incomes below 200% of poverty, births to mothers receiving Medi-Cal, births to mothers aged 18 and under, LBW births, percentage of late onset or no prenatal care, and infant mortality rate). To achieve an adequate sample of high-risk tracts, 200 tracts were selected from the high-risk stratum, and 100 tracts were selected from the low-risk stratum, for a total of 300 census tracts. In the second stage, births were sampled from within these tracts. We oversampled LBW births to ensure adequate samples for this population. The biggest strength of the resulting multilevel sample (MLS) is that it enables the use of multilevel analyses of the contextual determinants of adverse obstetric outcomes. The third stage comprised a supplemental sample of eligible women to create a final county sample (FCS) to be used as a routine surveillance tool. Essentially, the FCS (*n* = 6,274) included the MLS (*n* = 4,518) as well as an additional 1,746 women, who were randomly selected based on race-ethnicity and place of residence (SPA) to ensure that the final LAMB sample represented the entire 2007 live birth population in LAC as well as the racial diversity in each of the eight SPAs. The MLS dataset was used to examine research questions developed by the UCLA team, whereas the FCS was used to provide MCH surveillance information regarding LAC for the LACDPH team.

### 2.2. Measures

To develop the 2007 LAMB survey instrument, the UCLA team and the LACDPH teams reviewed the literature on obstetric outcomes and infant mortality from a life-course prospective, identified gaps in the available data in order to meet the needs of all interested stakeholders, conducted focus group meetings with new mothers and clinicians, and piloted the survey. The LACDPH conducted four focus groups with African-American (AA), Hispanic, White, and Asian/Pacific Islander mothers who had recently delivered live births in Los Angeles County. We learned that some women faced transportation barriers to attending prenatal care appointments. AA women felt that their health concerns were not taken seriously by providers, and many felt that they were treated as single welfare moms regardless of their marital or socioeconomic status. Clinicians shared concerns about women delaying their entry into prenatal care, the number of health issues that could not be solved in nine months of prenatal care, and the difficulty in getting their patients access to high-risk obstetrical care. Throughout the process, we involved community stakeholders in reviewing the survey questionnaire and survey procedures.

During early 2006, the LAMB pilot surveyed approximately 750 LAC mothers who met the eligibility criteria. Following careful review of the LAMB pilot, a few survey questions were revised to ensure that each survey would be completed within 30 minutes, a standard followed by other similar national surveys.

The final instrument covered over 80 prevalidated or modified versions of prevalidated questions, originating from widely used surveys, such as PRAMS, American Community Survey (ACS) [51], 2005 MIHA [4], National Maternal and Infant Health Survey (NMIHS) [48], and the Fragile Families Study (FFS) [44], as well as measures, including the Perceived Stress Scale (PSS) [38], Core Food Security Module (CFSM) [42], Adequacy of Prenatal Care Utilization (APNCU) Index [49], and Center for Epidemiological Studies-Depression (CES-D) [50]. The LAMB study also collected information on sociodemographic (e.g., annual household income and marital status) and acculturation (e.g., nativity, language spoken at home, and length of residence in the US) characteristics of mothers. A description of key measures for the 2007 LAMB is provided in Table 1. Based on the life-course framework, the LAMB survey instrument was organized to include the following topics: preconception health, prenatal care and maternal medical conditions during pregnancy psychosocial stress and resources during pregnancy, behavioral risk factors during pregnancy, and postpartum care and infant health. In addition, it also captured maternal demographic data. The final survey was translated into Spanish and Chinese, and a telephone translation service provided access in 88 languages. The survey was approved in 2007 by both LADPH and UCLA Institutional Review Boards.

### 2.3. Procedures

The LAMB study was conducted in four waves. A sample of over 4,000 eligible women (including the MLS and FCS) was drawn from the birth records once every three months during the 12-month survey period.  Table 2 displays the number of LAMB 2007 surveys sent and completed for each of our four racial-ethnic groups.

The 2007 LAMB study employed two modes of data collection: (1) mailed survey with multiple follow-up attempts for nonrespondents and (2) telephone interview for nonrespondents or respondents who requested the completion of the survey via telephone. This mixed-mode methodology has been used successfully by the National Maternal and Infant Health Survey (NMIHS) [48] and PRAMS. Each quarter of the project year, LACDPH followed the sampling method described earlier to identify eligible women from birth record data.

**Figure 1:**
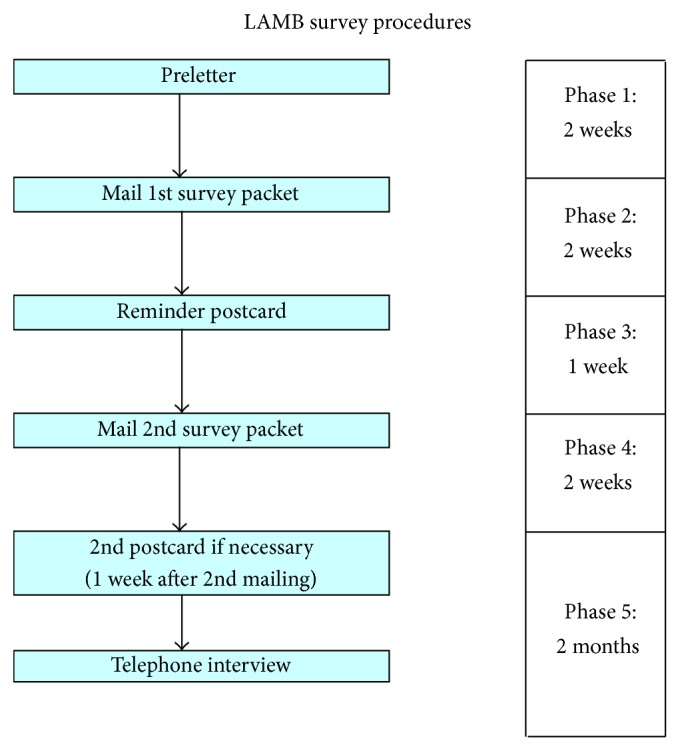
Diagram of 2007 LAMB survey procedures.

Potential respondents received an introduction letter and a survey packet, and nonrespondents received a reminder postcard, telephone follow-up, and reminder survey packet (Figure 1). A $20 gift certificate was mailed to each woman who completed the survey. For the telephone follow-up for nonrespondents, telephone numbers were obtained from Lexus Nexus. Up to eight telephone follow-up attempts were conducted by members of the LACDPH LAMB team for each nonrespondent and were administered at varying times and days of the week. Overall, surveys completed via telephone follow-up accounted for approximately 5% of the total number of completed surveys in our samples. Both the telephone attempts and the social marketing outreach improved response rates among those hardest to reach. Community outreach efforts included local faith-based organizations, WIC centers, Black Infant Health programs, and attendance at community gatherings to raise awareness of the project. At each gathering, in addition to distributing LAMB project pamphlets, trained LACDPH staff educated the participants about LAMB project and discussed what it means to each woman and her family, why we need mothers to complete the surveys, and how the findings may be used to improve health of mothers and babies. We concluded the meetings by encouraging each individual to actively spread the word about the LAMB survey.

To maintain a standardized data collection process, we developed comprehensive project protocols for survey mailing, survey review, data entry, and interviewing respondents. All project staff received standardized training by experienced staff and convened weekly to ensure that everyone followed survey protocols during the data collection period. The project protocols were revised based on findings from pilot to ensure project efficiency, higher response rate, and data quality.

### 2.4. Analytic Approach

Two sampling weights were calculated to account for differential selection and response probabilities—one for the FCS and one for the MLS. Both sampling weights followed a poststratification procedure with a slight difference. For the FCS, we used the raking procedure [53] to create weights in which the marginal totals of the weights aligned with the corresponding population totals of the characteristics used for selection. The raking procedure included seven population characteristics: LBW, PTB, SPA, maternal race, age, education, and nativity. For the MLS, race-ethnicity and maternal age were used to test for differential rates of initial participation. Since there were no differences between respondents and nonrespondents for these factors, nonresponse bias was not adjusted. SAS 9.2 was used to generate sampling weights.

## 3. Results

The MLS consisted of 4,518 women, whereas the FCS consisted of 6,264 women. The unadjusted response rates of the MLS and FCS were both 36% (Table 2). The adjusted response rate, which was computed according to the standards recommended by the American Association for Public Opinion Research [54] for the MLS and FCS, was 56%. We adjusted for reasons such as incorrect addresses and phone numbers (15%), loss to follow-up (could not locate or moved to a different place; 4%), language issues (1%), and maternal deaths (0.5%).

Descriptive analyses of the 2007 FCS were published in an online report entitled the* 2007 LAMB surveillance report* [55], which identifies health disparities for women by race/ethnicity and geographic areas in an effort to determine which communities and health indicators require more immediate attention. For example, in examining the indicators for preconception health and health access, Hispanics were more likely than any other group to be uninsured prior to pregnancy. Disparities in these factors were often related to increased subsequent health risks and illness. Similarly, uninsured women reported more difficulty accessing medical care and had lower rates of receiving preventive health screenings compared to insured women.

Table 3 provides a comparison of the weighted percentage distributions of selected maternal characteristics and obstetric outcomes among the MLS and FCS samples. Overall, women in both samples were more likely to be Hispanic, aged 20–34 years, completed at least 12 years of schooling, had an annual household income of less than $39,000, and primarily spoke English. In general, the MLS had larger numbers of Hispanic, less educated, low-income, unmarried, and Spanish-speaking women when compared to the FCS.

## 4. Discussion

The 2007 LAMB study has a number of unique strengths that contribute to the field by taking MCH surveillance and research to the next level. The LAMB study represents a successful collaboration between an academic institution (UCLA) and a local public health department (LACDPH). The complexity of maternal and infant health problems has often made them unsuited to the traditional* outside-expert-driven* research and intervention approaches. Before LAMB, LACDPH struggled to describe health and demographic trends or correlations pertaining to birth outcomes due to lack of comprehensive, quality perinatal health data. This collaboration led to the creation of a theoretically based research dataset as well as a community surveillance system that highlights the importance of an alternative paradigm for MCH research. Several manuscripts using LAMB study findings have been published by peer-reviewed journals and have explored a wide range of MCH topics, including the development of an index to operationalize women's resources during pregnancy (i.e., personal capital) [56] and its relationship with stress during pregnancy [57], unintended births among Mexican women in LAC [58], and predictors and barriers to postpartum care [59]. Further, the uniqueness of the LAMB study sampling methods allows researchers to perform multilevel analysis by linking the LAMB study dataset with census data to better understand adverse obstetric and birth outcomes; several manuscripts are currently in progress that have employed such multilevel analysis [60, 61].

Further, the LAMB study serves as an example for other counties for how to build a local surveillance and monitoring system to better address serious public health issues such as LBW and PTB. Following preestablished scientific methods, LACDPH is expanding the existing LAMB surveys system by implementing a follow-up project which reinterviews LAMB mothers when babies reach two years of age.

Additionally, as LAC communities were involved from the onset of the study, it is more likely that the LAMB study findings will be employed to improve local policies and programs that affect the health of the populations that they serve. Since the LAMB project began, LAMB findings have helped the LAC public health community to improve birth outcomes by focusing interventions on policy issues identified by the study, such as preconception health, perinatal mental health, racism, and healthy weight for pregnant women. MCAH and community stakeholders have utilized LAMB data in a myriad of ways. Data on pregnancy weight gain have supported health promotion efforts for the Healthy Weight for Women of Reproductive Age Action Learning Collaborative and tool development for worksite wellness programs. Also, data on vitamin supplementation and reproductive health services have assisted LAC in integrating components of preconception health into existing local public health and related programs. Additionally, data demonstrating the importance of identifying depressed mood during pregnancy among Latino and African-American women in LAC were used to support the formation of the Los Angeles Perinatal Mental Health Task to improve the mental health of perinatal women. Examples of other LAC accomplishments that were driven by collaborations with LAMB community partners include (1) training Comprehensive Perinatal Services Program providers on breastfeeding and perinatal depression curricula and (2) forming the Black Infant Health (BIH) initiatives to focus on healthy lifestyles and prevent high-risk behaviors among African-American mothers. These successes have also been highlighted in a report from the Health Resources and Services Administration's MCH Bureau on Implementing a Life Course Framework [62].

Moreover, among other important findings, our study established that adverse obstetric outcomes, including LBW and PTB, are a major public health concern affecting LAC mothers and infants. Therefore, the LAMB study has enabled LACDPH to collect and disseminate comprehensive data on the biomedical, psychosocial, and behavioral risk factors for poor obstetric outcomes as well as the maternal resources (both internal and social) that may improve birth outcomes and reduce racial and ethnic disparities in these outcomes. Additionally, the LAMB study has incorporated the life-course approach by examining the risks and resources occurring in the preconception, prenatal, and postpartum periods. By using the life-course approach, the results that emerge will allow us to draw conclusions and understand issues that are most relevant during critical periods of life and affect the most vulnerable populations.

Despite its many contributions, the LAMB study also has some potential limitations. First, its cross-sectional nature precludes the assumption of causality between predictors and outcomes. Second, because the survey was completed postpartum, responses to questions about the preconception and prenatal periods may have been subject to recall bias. Third, the LAMB study had a relatively low response rate, though we argue that LAMB study findings support national findings on key indicators; for example, the percentage of breastfed infants in LAC was 85% in 2007, similar to 75% of infants born in 2007 nationwide [63].

## 5. Conclusions

The LAMB study represents the successful surveillance collaboration between an academic institution and a local public health department. Overall, the unique contributions of the 2007 LAMB study are attributable not only to its inclusion of an ethnically and racially diverse study sample but also to the sampling design with which these data were collected. By using a multilevel sampling plan, multilevel analyses can be conducted to elucidate the differences between high- and low-risk areas in LAC and how the environments play a role in the development of outcomes for different areas. Observed individual differences can be placed in the context of these factors and assist policymakers to make better decisions for the populations that they serve, as well as develop effective programs to address the needs of the diverse individuals in the community. While the results from the LAMB study cannot be formally generalized to the US on the whole, LAC represents a diverse, metropolitan center that is as large as some states in the US. Therefore, LAMB study findings can help inform decision-making and program development around the country in similar metropolitan areas to address the existing health disparities in obstetric outcomes for these vulnerable populations. Due to the successful experience of the LAMB project, the project has now become a routine surveillance tool for LACDPH to collect MCH data every other year.

## Figures and Tables

**Table 1:** Overview of key measures and data sources in 2007 LAMB survey.

Time period	Variable	Measures/data sources
Preconception variables	Insurance status and type of insurance	Center of Disease Control and Prevention's (CDC's) Pregnancy Risk Assessment Monitoring System (PRAMS) [5]; LAMB study team
	Interaction with healthcare professional to prepare for pregnancy	LAMB study team
	Content of preconception care counseling	LAMB study team
	Health conditions	PRAMS; LAMB study team
	Tobacco use	LAMB study team
	Folic acid/multivitamin use	Modified from PRAMS
	Pregnancy intention and partner pregnancy intention	Modified from PRAMS
	Birth control and emergency contraception use	Modified from PRAMS; LAMB study team
	Gravidity and parity	LAMB study team
	Previous pregnancy and birth outcomes	PRAMS; LAMB study team

Pregnancy variables	Maternal stress (i.e., perceived stress and stressful life events)	9-item version of Cohen's Perceived Stress Scale (PSS) [38]; PRAMS Life Events List
	Maternal internal resources (i.e., self-esteem and mastery)	3-item Rosenberg short form [39]; 4-item Pearlin short form [39]
	Job strain	Subscale of Karasek's Job Content Questionnaire [40]; unemployment rate item from ACS
	Maternal behaviors (i.e., smoking, alcohol use, and drug use)	PRAMS; 2005 California Maternal and Infant Health Assessment (MIHA) [4]; National Survey of Family Growth (NSFG) [41]
	Food insecurity	Core Food Security Module (CFSM) [42]
	Partner conflict	Marital Strain Scale (MSS) [43]
	Partner support/involvement	Fragile Families Study (FFS) [44]; Early Head Start Evaluation [45]
	Partner violence	Abuse Assessment Screen (AAS) [46]
	Social network support	PRAMS
	Racial discrimination	Developed by Krieger (1990) [47] and modified by Collins and David (1997) [18] to assess pregnancy and lifetime exposures to interpersonal racial discrimination
	Healthcare content, access, and quality	Structure was measured by access and availability of services with items developed by the LAMB study team; process was measured by content with items from National Maternal and Infant Health Survey (NMIHS) [48]; adequacy of prenatal care was measured with items from the Adequacy of Prenatal Care Utilization (APNCU) Index [49]; commuting characteristics items from ACS
	Pregnancy complications	PRAMS; LAMB study team; 2007 California birth certificate data
	Neighborhood support	Project on Human Development in Chicago Neighborhoods (PHDCN) [19]
	Neighborhood services	LAMB study team; availability of parks, crime rate, and mortgage status items from ACS

Postpartum variables	Birth outcomes (LBW, PTB)	California birth certificate data
	Quality of healthcare during delivery	Item developed by LAMB study team
	Breastfeeding	Modified from PRAMS; LAMB study team
	Baby sleeping pattern/cosleeping	PRAMS; LAMB study team
	Well-baby checkup	LAMB study team
	Postpartum checkup	PRAMS; LAMB study team
	Partner violence	Abuse Assessment Screen; ACS
	Postpartum depression	Modified from Center for Epidemiological Studies-Depression (CES-D) [50]
	Contraception use	PRAMS

General: demographic data	Annual household income	LAMB study team
	Median household income	American Community Survey (ACS) [51]
	Immigration (i.e., length of residence in the US)	LAMB study team
	Acculturation (i.e., language spoken at home, nativity)	ACS
	Maternal education	2007 California birth certificate data
	Race/ethnicity	2007 California birth certificate data
	Marital status with baby's father	PRAMS
	Occupation	Census data

**Table 2:** Comparison of 2007 LAMB surveys sent and completed by race-ethnicity among the multilevel (MLS) and final county (FCS) samples (crude response rate).

Race/ethnicity	Number of women sampled	Number of completed surveys returned	Crude response rate (%) (number of completed surveys/number of women sampled)
Total			
MLS	12,675	4,518	35.6%
FCS	17,570	6,264	35.7%
Non-Hispanic White			
MLS	2,087	880	42.2%
FCS	3,084	1,308	42.4%
Non-Hispanic Black			
MLS	937	272	29.0%
FCS	3,244	987	30.4%
Hispanic			
MLS	8,038	2,760	34.3%
FCS	8,319	2,887	34.7%
Asian/Pacific Islander			
MLS	1,509	557	36.9%
FCS	2,686	987	36.7%

**Table 3:** Weighted percentage distribution of selected maternal characteristics and birth outcomes in 2007 LAMB study.

	Multilevel sample (MLS) (%)	Final county sample (FCS) (%)
Total		
Unweighted	*N* = 4,518	*N* = 6,264
Weighted	*N* = 151,813	*N* = 151,813
Race/ethnicity		
Non-Hispanic White	12.4	16.9
Hispanic	74.1	63.1
Non-Hispanic Black	4.5	7.5
Asian/Pacific Islander	8.3	11.2
Age		
<20	11.0	9.4
20–24	22.8	22.1
25–34	50.0	50.0
35+	17.3	18.5
Mother's years of education		
<12	37.8	31.7
=12 (high school diploma or GED)	25.5	25.8
>12	36.7	42.5
Marital status (during delivery)		
Married	52.9	55.6
Not married	47.1	44.4
Language(s) usually spoken at home		
English	60.9	65.4
Spanish	59.8	50.5
Asian language	5.5	7.0
Other	3.5	4.9
Annual household income		
<$20,000	46.0	41.0
$20,000–$39,999	22.1	21.8
$40,000–$59,999	8.4	9.0
$60,000–$99,999	9.6	11.0
$100,000 and more	8.7	11.0
Birth outcomes		
Preterm birth	12.2	11.4
Low birth weight	8.0	7.4
